# A Protective Role of Arecoline Hydrobromide in Experimentally Induced Male Diabetic Rats

**DOI:** 10.1155/2015/136738

**Published:** 2015-01-28

**Authors:** Indraneel Saha, Joydeep Das, Biswaranjan Maiti, Urmi Chatterji

**Affiliations:** Department of Zoology, University of Calcutta, 35 Ballygunge Circular Road, Kolkata 700 019, India

## Abstract

*Objectives.* Arecoline, the most potent and abundant alkaloid of betel nut, causes elevation of serum testosterone and androgen receptor expression in rat prostate, in addition to increase in serum insulin levels in rats, leading to insulin resistance and type 2 diabetes-like conditions. This study investigated the role of arecoline on the reproductive status of experimentally induced type 1 diabetic rats.* Methods. *Changes in the cellular architecture were analyzed by transmission electron microscopy. Blood glucose, serum insulin, testosterone, FSH, and LH were assayed. Fructose content of the coagulating gland and sialic acid content of the seminal vesicles were also analyzed.* Results.* Arecoline treatment for 10 days at a dose of 10 mg/kg of body weight markedly facilitated *β*-cell regeneration and reversed testicular and sex accessory dysfunctions by increasing the levels of serum insulin and gonadotropins in type 1 diabetic rats. Critical genes related to *β*-cell regeneration, such as pancreatic and duodenal homeobox 1 (*pdx-1*) and glucose transporter 2 (*GLUT-2*), were found to be activated by arecoline at the protein level.* Conclusion.* It can thus be suggested that arecoline is effective in ameliorating the detrimental effects caused by insulin deficiency on gonadal and male sex accessories in rats with type 1 diabetes.

## 1. Introduction

In a population-based study, betel nut chewing has been associated with an increase in serum insulin levels and a higher risk of type 2 diabetes mellitus [1]. Elevated insulin levels are known to reduce biological responses, leading to insulin resistance and subsequently glucose intolerance, endothelial dysfunction, elevated inflammatory markers, cardiovascular disease, hypertension, and certain forms of cancer [2, 3]. These reports confirm that consumption of betel nuts leads to metabolic disorders that may eventually increase the risk of type 2 diabetes, along with hypoglycemia, in chronic users. However, till date, there is no report of betel nut chewing being associated with or increasing the risk of type 1 diabetes and associated hyperglycemia, in men.

It is a well-established fact that serum insulin levels have a profound influence on the male reproductive physiology [4]. LH and testosterone concentrations are known to decrease under hypoglycemic conditions, caused by increase in serum insulin levels, even though dehydroepiandrosterone (DHEA) concentrations increased during hypoglycemia [4]. Thus, hypoglycemia not only has a suppressive effect on gonadal steroidogenesis but also suppresses testosterone secretion. On the other hand, assessment of the effect of hyperglycemia on male fertility in rats revealed that animals injected with streptozotocin to induce diabetes also showed significantly lowered serum testosterone level, decreased epididymal weight, and diminished sperm count compared with buffer-injected controls [5]. Diabetes also induced significant reduction in mating behavior and had significantly diminished reproductive organ weight, testicular sperm content, epididymal sperm content, and sperm motility and decreased in vitro testosterone secretion relative to the control [6]. Alloxan-induced diabetes led to a decrease in the body and prostatic weights, as well as variations in prostate morphology and stereology, including intense epithelial atrophy combined with chronic inflammation and premalignant lesions, with high levels of cellular proliferation and nuclear atypia [7]. In addition, even maternal hyperglycemia has deleterious effects on testicular parameters during fetal life and significantly decreases serum testosterone levels of offspring [8].

Arecoline, which is the most active chemical compound of betel nut [9] and constitutes up to 0.8% by weight of the ripe nut [10] or 7.5 mg/g weight [11], has been found to increase serum insulin levels in normal rats [12], which conforms to previous reports. With regard to the effects of arecoline on the male reproductive organs and hormonal levels of normal rats, studies in our laboratory have shown that arecoline stimulates testicular functions and enhances testosterone secretion with an augmented expression of androgen receptors in the ventral prostate [13], quite contrary to the suppressive effects of increased insulin levels in animals. Thus, based on the stimulatory property of arecoline, we attempted to investigate whether arecoline can restore the serum insulin and testosterone levels in experimentally induced type 1 diabetic rats, where low serum insulin levels deregulate the gonadal and prostate physiology. Consequently, in this study, we have summarized the effects of arecoline administration on serum insulin levels in alloxan-induced diabetic rats and its consequence on the circulating testosterone levels and sex accessory glands in male Wistar rats. This study is the first to elucidate the effects which regular betel nut chewing may have on the reproductive physiology of men with chronic type 1 diabetes and a possible mechanistic explanation for alterations by arecoline.

## 2. Material and Methods

### 2.1. Animal Model

Adult male Wistar rats (~100 gm body wt) were collected from the breeding colony and were housed in polythene cages at a temperature of 25°C with a regular light-dark cycle (12L : 12D) with standard diet. Rats were ~100 days old and sexually mature when experiments commenced [14]. Food and water were given* ad libitum* for 5 days for acclimatization before commencement of the experiments. Animal experiments were carried out following the “Principles of Laboratory Animal Care” (NIH Publication number 85-23 revised in 1985). This study was carried out in strict accordance with the recommendations in the Guide for the Care and Use of Laboratory Animals of the Indian Laws of Animal Protection and the protocol was approved by the Committee on the Ethics of Animal Experiments of the University of Calcutta (IAEC number 885/ac/05/CPCSEA dated 25.2.2005). All surgery was performed under sodium pentobarbital anesthesia, and all efforts were made to minimize suffering. Five rats (*n* = 5) were taken in each experimental group.

### 2.2. Arecoline Administration

Arecoline hydrobromide (methyl-1-methyl-1,2,5,6-tetrahydronicotinate; Sigma, USA), dissolved in normal saline (0.9% NaCl), was injected intraperitoneally at a dose of 10 mg/kg body weight for 10 days, as determined previously as the optimum dose [13]. Each dose (1 mg/100 gm body wt) was divided equally into half (0.5 mg/100 gm body wt), and each half dose was injected twice daily (11 a.m. and  5 p.m.) because of its short half-life [15].

### 2.3. Induction of Diabetes and Treatment Groups

Alloxan, a potent diabetogenic drug (Sigma, USA), was dissolved in citrate phosphate buffer, pH 7, and injected intraperitoneally at a dose of 75 mg/kg body weight once daily for 10 days to induce experimental diabetes. The experimental groups were divided as follows: (i) Group A served as the control for diabetic rats and received citrate phosphate buffer; (ii) Group B received 7.5 mg/100 gm alloxan injection; (iii) Group C served as control for arecoline treatment and received normal saline; (iv) Group D received arecoline at 1 mg/100 gm body weight; and (v) Group E received alloxan for 10 days, followed by treatment with arecoline for another 10 days. Each group consisted of five animals (*n* = 5) and all experiments were performed in triplicate.

### 2.4. Transmission Electron Microscopy

Processing for electron microscopy and analysis were done according to the method of Dasgupta et al., 2010 [16]. Testes and prostate glands were dissected out and trimmed free of fat. The tissues were cut into small pieces (~1 mm^3^) and fixed in 2.5% glutaraldehyde and 1% paraformaldehyde in 0.1 M phosphate buffer (pH 7.4) for 6 to 8 h at 4°C. After washing in buffer, the tissue samples were postfixed in 1% osmium tetroxide for 2 h at 4°C. Tissues were then dehydrated through ascending grades of ethanol, infiltrated, and embedded in araldite CY 212. Thin sections (60–80 nm) were contrasted with uranyl acetate and alkaline lead citrate and viewed under a Morgagni 268D transmission electron microscope (Fei Company, The Netherlands) at an operating voltage of 80 KV. For all specimens, digitized images of cellular organelles (*n* = 20 for each specimen) were recorded at a magnification of 28000x.

### 2.5. Biochemical Assays

All experiments were terminated on Day 11. Serum was isolated from the rats under fasting conditions and stored at −20°C until assayed for insulin, glucose, and testosterone. The coagulating gland and seminal vesicles were dissected, weighed in a semimicroanalytical balance (Mettler, Switzerland), and stored at −20°C for sialic acid and fructose assays.

### 2.6. Estimation of Serum Insulin

Serum insulin was quantified using the EIA kit (DSL, UK) according to the method of O'Rahilly and Moller, 1992 [17]. In brief, the serum samples were incubated with anti-insulin antibody conjugate in microtitration wells and coated with anti-insulin antibody. After incubation and washing, the wells were incubated with tetramethylbenzidine (TMB) as the substrate. 0.2 M sulphuric acid was used to stop the reaction and the degree of enzymatic turnover of the substrate was determined by dual wavelength absorbance at 450 and 620 nm.

### 2.7. Estimation of Blood Glucose

Blood glucose levels of the different treatment groups were measured by the glucose oxidase-peroxidase (GOD-POD) enzymatic method of Trinder, 1969 [18], using the Autospan kit (Span Diagnostic Ltd., India). Glucose was first oxidized to gluconic acid and hydrogen peroxide by glucose oxidase. In a subsequent peroxidase-catalyzed reaction, the oxygen liberated was accepted by the chromogen system to give a red coloured quinoneimine compound. The absorbance was measured at 505 nm (Smart Spk 3000, BioRad, Australia). The intensity of the red colour was directly proportional to the concentration of glucose present in the sample.

### 2.8. Intraperitoneal Glucose Tolerance Test (IPGTT)

Normal, arecoline-treated, diabetic, and diabetic-arecoline-treated rats were subjected to IPGTT. Rats were fasted overnight (16 ± 2 hours) and fasting blood glucose was measured in the rats using a hand-held glucometer (ACCU-CHECK, Roche, Germany). After measuring the baseline fasting blood glucose at time = 0 minutes, a glucose challenge was administered (1 g/kg, i.p.), marking the start of the IPGTT, together with administration of arecoline. Blood glucose was determined every 30 minutes in a drop of blood from the tail for the next 2 hours [19].

### 2.9. Estimation of Liver Glycogen Content

Liver glycogen levels were measured by the method of Hassid and Abraham, 1957 [20]. Liver tissues were collected in 30% KOH solution and boiled in water bath for 30 min. Next, 0.5 mL of saturated sodium sulfate was added and glycogen was precipitated by the addition of 1.2 mL of 95% ethanol. The tubes were heated to boil, cooled, and centrifuged at 3000 rpm for 10 min. The mother liquor was decanted and precipitated, and glycogen was redissolved in 2 mL of distilled water, precipitated again with 2.5 mL of 95% ethanol. The precipitate was cooled, diluted in water in a volumetric flask, and vortexed. Glycogen solution was further diluted with water in a separate volumetric flask to yield glycogen concentration of approximately 3 to 30 r/mL. Five mL of the aliquot, equivalent to 15 to 150 r of glucose, was taken in a separate tube. The other tube contained 5 mL of water and served as blank. The tube containing 5 mL of glucose (10 r of hexose) served as standard. All the tubes were cooled and 10 mL of 0.2% anthrone reagent (1.2 g anthrone in 100 mL of 95% sulfuric acid) was added to each tube and heated for 10 min. Finally, samples were cooled and O.D. was recorded at 620 nm by a spectrophotometer (Shimadzu). The amount of glucose was converted to glycogen by dividing with the factor 1.11.

### 2.10. Estimation of Serum Testosterone, FSH, and LH

Serum testosterone, FSH, and LH levels were assayed by ELISA using the pathozyme testosterone kit (Omega, UK, OD497) and Eliscan FSH and LH kits [13]. Goat anti-rabbit IgG-coated wells were incubated with serum of arecoline-treated and untreated rats; testosterone, FSH, and LH standards; and rabbit anti-testosterone, anti-FSH, and anti-LH reagents, respectively. Unbound hormones were then removed, followed by addition of hormone-HRP conjugate reagent. Tetramethylbenzidine (TMB) solution was added as the substrate and colour development stopped by adding dilute sulfuric acid. Absorbance was measured by a Qualigen Plate Reader (PR-601, UK) at 450 nm. The testosterone, FSH, and LH concentrations of the untreated and treated serum were run concurrently with the standards and calculated from the standard curve, obtained by plotting the concentration of the standards versus absorbance. Specific cross-reactivity was observed at 75% level. Coefficients of intra- and interassay variations were recorded at 5% and 8%, respectively.

### 2.11. Estimation of Fructose

Fructose concentration of the coagulating gland was assayed according to the method described by Roe et al., 1949 [21]. Briefly, the coagulating gland was weighed and homogenized in 5 mL distilled water. The homogenate was centrifuged at 8000 ×g for 5 min at 4°C. One mL of the supernatant was added to 1 mL of resorcinol reagent and 7 mL of 30% HCl, and the mixture was heated in an 80°C water bath for 10 min. The reaction mix was cooled to room temperature and the optical density was measured by a spectrophotometer (PerkinElmer) at 520 nm.

### 2.12. Estimation of Sialic Acid

Sialic acid content of the seminal vesicle was assayed from the homogenate of the arecoline-treated and untreated seminal vesicles of experimentally induced diabetic and nondiabetic rats [22]. The extracts were oxidized with sodium periodate in concentrated phosphoric acid. The periodate oxidation product was coupled with thiobarbituric acid and the resulting chromophore was extracted using cyclohexanone. The absorption maximum for sialic acid was measured at 549 nm. A second absorption maximum was also measured at 532 nm, to assess the presence of 2-deoxyribose. The correction was made by subtracting the data at 532 nm from the data at 549 nm.

### 2.13. Western Blot Analysis

Pancreas from treated and untreated animals was collected and protein isolated in ice-cold RIPA Buffer (150 mM NaCl, 50 mM Tris, 0.1% Triton X-100, and 0.1% SDS) containing protease inhibitors [4-(-2-aminoethyl)benzenesulfonyl fluoride), EDTA, leupeptin, aprotinin, and bestatin] and assayed by the Bradford method. 40 *μ*g of pancreatic protein was loaded onto a 12% SDS/PAGE gel. Proteins were transferred to a PVDF membrane and probed with rabbit anti-pdx-1 (1 : 1000) and rabbit anti-GLUT-2 (1 : 1500) for 1 h. Blots were rinsed three times in PBS and incubated with anti-rabbit horseradish peroxidase-conjugated secondary antibody (1 : 2000 in 5% nonfat dried milk). Following a second series of washes, the proteins were visualized by staining with 3,3′-diaminobenzidine, followed by densitometric analysis on a BioRad Gel Documentation System.

### 2.14. Statistical Analysis

All individual experiments were carried out three times independently in order to ensure repetition of results. All data were expressed as mean ± SEM. Data were analyzed statistically by one way analysis of variance followed by Tukey's post hoc test and Student's *t*-test [23] to ascertain the degree of significance between experimental groups.

## 3. Results

### 3.1. Arecoline Administration Ameliorated Serum Insulin Levels and Attenuated Blood Glucose Levels of Alloxan-Treated Rats

**Figure 1:**
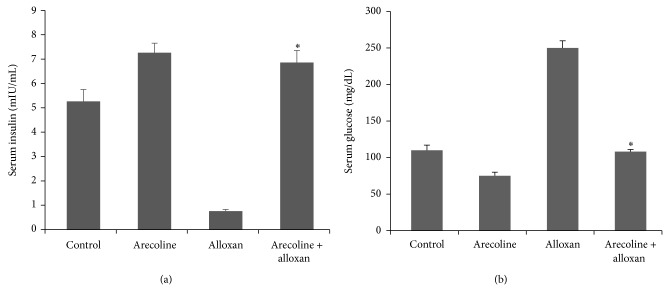
Effect of arecoline on serum insulin and glucose levels in normal and alloxan-induced diabetic rats. (a) Arecoline treatment of normal and alloxan-treated rats compared to diabetic rats indicated differential insulin expression. (b) Blood glucose levels in alloxan-treated normal and diabetic rats, as compared to the control rats. All assays were done in triplicate and each value is represented as mean ± SEM, ^*^ *P* < 0.01.

As expected in the diabetic control, there was severe hyperglycemia as compared to the normal animals. Alloxan treatment significantly lowered (*P* < 0.01) the serum insulin levels of treated rats as compared to the control rats. Since the results for the control animals which received either citrate phosphate buffer or normal saline were similar, a single representation has been shown in all subsequent experiments. However, compared to the diabetic control, arecoline treatment increased the level of insulin in both alloxan-induced diabetic rats and the normal rats which received arecoline injections only (Figure 1(a)). Arecoline could recover the level of insulin in experimentally induced diabetic rats to values that are observed in the control animals. The interassay variance was 4% and intra-assay variance was 5%. The blood glucose level, which had increased in alloxan-treated animals, was simultaneously lowered by arecoline treatment in both normal and diabetic rats (Figure 1(b)). The interassay variance was 3% and intra-assay variance was 6%.

### 3.2. IPGTT

**Figure 2:**
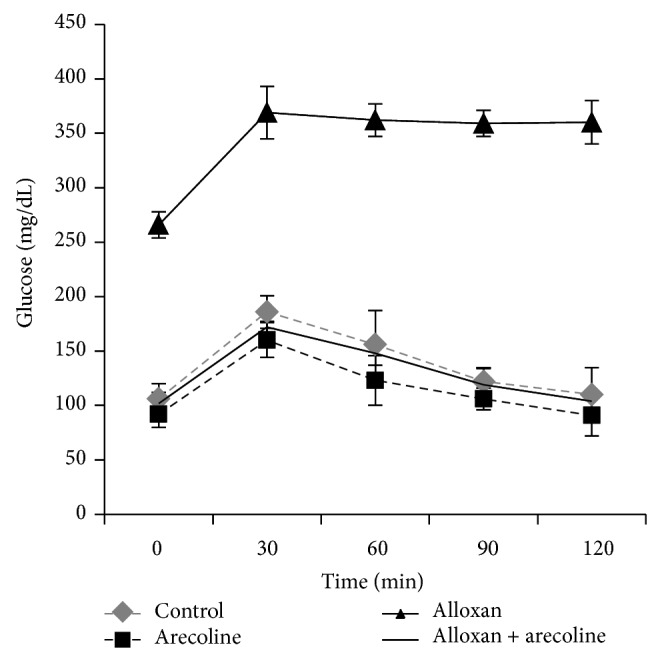
Intraperitoneal glucose tolerance curve (IPGTC) for control, arecoline, alloxan, and arecoline + alloxan-treated groups. Blood glucose level was measured at times 0, 30, 60, 90, and 120 min after giving 1 g/kg of glucose orally. All assays were done in triplicate and each value is represented as mean ± SEM, ^*^ *P* < 0.01.

The effect of arecoline extract on GTT has been summarized in Figure 2. The comparison of GTT plots for control, arecoline, and arecoline-treated diabetic groups may suggest a relative improvement of insulin sensitivity and a reduction of blood glucose levels in the arecoline-treated groups. The change further supports the ability of arecoline to stimulate insulin secretion from pancreatic beta cells.

### 3.3. Effect on Liver Glycogen Content

The hepatic glycogen content in diabetic rats decreased sharply as compared to control animal (Table 1). After administration of arecoline to diabetic rats, a significant increase (*P* < 0.01) in liver glycogen content as compared to diabetic control group was observed.

### 3.4. Arecoline Recovered Circulating Testosterone Levels in Alloxan-Induced Diabetic Rats

**Figure 3:**
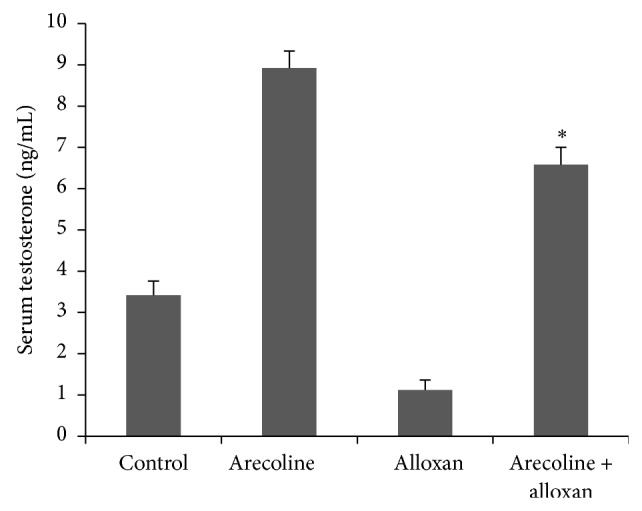
Effect of arecoline on serum testosterone levels in normal and alloxan-induced diabetic rats. Normal and alloxan-induced diabetic rats were treated with arecoline. Arecoline treatment of diabetic rats significantly altered the levels of testosterone. All assays were done in triplicate and each value is represented as mean ± SEM, ^*^ *P* < 0.01.

It is well established that the serum testosterone levels are significantly diminished in experimentally induced diabetic rats. In contrast, it has also been observed that arecoline treatment upregulates testosterone concentration in a dose- and time-dependent manner in normal rats. Consequently, we analyzed the testosterone levels in rats treated with alloxan. Interestingly, arecoline administration, which increased the testosterone levels in normal rats, also augmented the testosterone levels in the alloxan-treated diabetic rats (Figure 3) and, hence, could significantly (*P* < 0.01) recover the lowering of testosterone levels in diabetic rats. The interassay variance for testosterone is 3% and intra-assay variance is 5%.

### 3.5. Arecoline Elevated Serum FSH and LH Levels in Alloxan-Induced Rats

**Figure 4:**
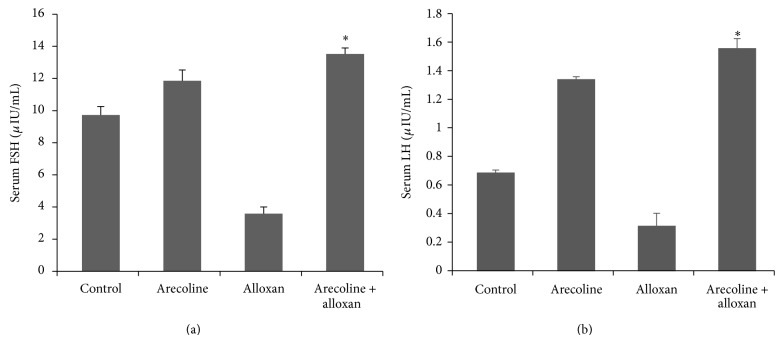
Effect of arecoline on the levels of serum gonadotropins in normal and alloxan-induced diabetic rats. ELISA analysis of serum FSH (a) and LH (b) levels in alloxan-induced diabetic rats compared to the control animals and arecoline-treated diabetic rats. All assays were done in triplicate and each value is represented as mean ± SEM, ^*^ *P* < 0.01.

Since arecoline significantly increased the levels of serum testosterone, we next investigated its effects on the gonadotropins, as they are the chief components upstream of androgen biosynthesis and determine the production of the male steroid. Although levels of both serum FSH (Figure 4(a)) and LH (Figure 4(b)) were reduced (*P* < 0.01) in alloxan-induced diabetic rats compared to the control animals, arecoline treatment significantly increased (*P* < 0.01) the levels of both gonadotropins in untreated and in diabetic rats. Coefficients of intra-assay and interassay variations for FSH were 5% and 7% and for LH they were 6% and 8%, respectively.

### 3.6. Recovery of Leydig Cell Ultrastructure by Arecoline

**Figure 5:**
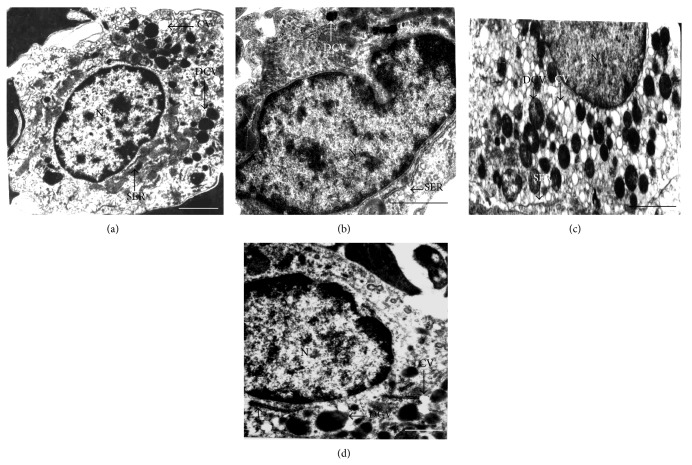
Transmission electron micrographs of arecoline-treated and untreated Leydig cells. (a) Untreated rats showing ovoid euchromatic nucleus (N) with smooth endoplasmic reticulum (SER), dense core vesicles (DCVs), and a few clear vesicles (CVs). (b) Arecoline-treated animals showing enlarged nucleus (N) with abundance of SER, DCVs, and CVs. (c) Alloxan treatment of rats showed indented hyperchromatic and pycnotic nucleus (N) with scanty SER and DCVs in the Leydig cells. (d) Arecoline treatment of alloxan-induced diabetic rats indicating hyperactive Leydig cells with enlarged nucleus (N), conspicuous SER, and several DCVs. Scale bars: 1 *μ*m (a, b, and d) and 1.5 *μ*m (c).

The Leydig cells are the major targets for the gonadotropins in male rats, as well as the site of testosterone synthesis. Since arecoline induced expression of both LH/FSH and testosterone in control rats and could significantly recover their levels to values observed for control animals in the alloxan-induced diabetic rats, we investigated the effect of arecoline on the ultrastructure of Leydig cells. Electron microscope studies showed that the control Leydig cells contained ovoid euchromatic nucleus with moderate number of smooth endoplasmic reticula (SER), abundance of dense core vesicles (DCVs), and few clear vesicles (CVs) (Figure 5(a)). In contrast, Leydig cells of the alloxan-treated rats showed few SER, mitochondria (M), scanty DCVs, and hyperchromatic pycnotic nucleus with indented nuclear membrane (Figure 5(b)). Leydig cells of arecoline-treated rats showed enlarged nucleus with abundance of SER, DCVs, and CVs (Figure 5(c)). In arecoline-treated diabetic rats, Leydig cells showed enlarged nucleus with conspicuous SER and DCVs (Figure 5(d)), cellular characteristics that are comparable to those seen in the Leydig cells of control rats. Quantification of the data is summarized in Table 2. Hence, arecoline treatment could overcome the degenerative changes brought about by alloxan-induced diabetes in the Leydig cells of the rats, conforming to the recovered levels of serum testosterone.

### 3.7. Effect on Fructose and Sialic Acid Content

**Figure 6:**
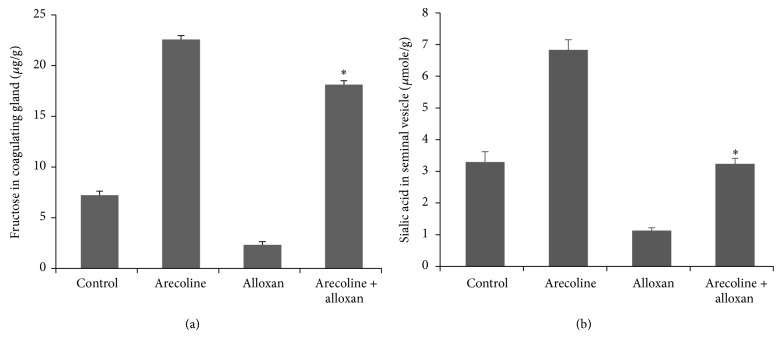
Effect of arecoline on fructose content of coagulating glands and sialic acid content of seminal vesicles in normal and alloxan-induced diabetic rats. Fructose contents of the coagulating glands (a) and sialic acid contents of seminal vesicles (b) in diabetic rats and after arecoline treatment of normal and diabetic rats. All assays were done in triplicate and each value is represented as mean ± SEM, ^*^ *P* < 0.01.

The role of testosterone in the maintenance of the male accessory reproductive organs has been well demonstrated. Conventional bioassays thus help in evaluating the potency of the hormone, since formation of fructose and sialic acid in the accessory reproductive organs of the male is directly dependent on androgenic activity [24, 25]. Our results have confirmed that both fructose (Figure 6(a)) and sialic acid (Figure 6(b)) contents of the coagulating gland and seminal vesicle, respectively, decreased after alloxan treatment compared to control rats, possibly as a downstream effect of reduced serum testosterone. Arecoline treatment, which was seen to elevate fructose and sialic acid concentrations in normal rats, could also enhance the concentration of sialic acid of alloxan-induced diabetic rats to the level observed in control animals and the concentration of fructose to almost that observed in arecoline-treated normal rats (almost 3-fold higher than control levels; *P* < 0.01). The interassay variance for sialic acid and fructose was less than 7%.

### 3.8. Effect on Ultrastructure of Ventral Prostate Epithelium

**Figure 7:**
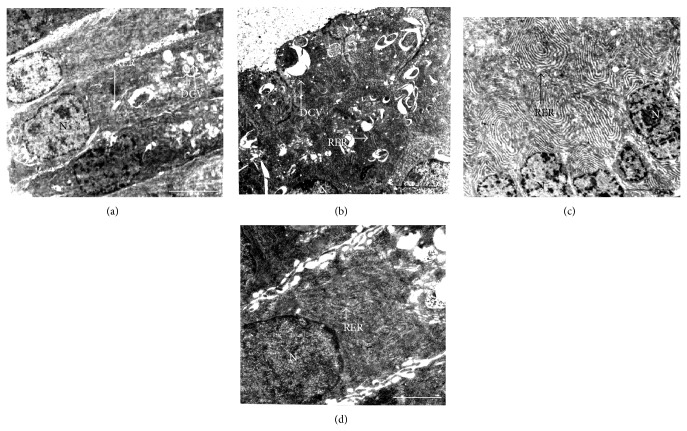
Transmission electron micrographs of arecoline treatment in the prostatic cells of the rats. (a) Ultrastructure of the prostate of untreated rats showing ovoid euchromatic nucleus (N) and moderate number of rough endoplasmic reticula (RER). (b) Arecoline treatment led to an abundance of well-organized RER in the epithelial cells of the prostate. (c) Alloxan-induced diabetic prostate with reduced nuclear size (N) and increased degenerated dense core vesicles (DCVs) and RER in the condensed cell cytoplasm. (d) Arecoline treatment of alloxan-induced rats showing enlarged nucleus and abundance of RER. Scale bars: 1 *μ*m (a, b, and c) and 1.5 *μ*m (d).

Since the development and function of the prostate gland are also under direct influence of androgens and we have reported earlier that arecoline treatment leads to hyperactivity and increased cellular proliferation of the prostate gland of normal rats [13], the effect of arecoline on the ultrastructure of the prostate gland was investigated under diabetic conditions. The results indicated that the ventral prostate of control animals showed oval euchromatic nucleus with moderate number of RER and DCVs (Figure 7(a)), whereas alloxan treatment caused prominent degenerative changes in the epithelial cells of the ventral prostate with indistinguishable cell membranes, RER, and mitochondria. The cytoplasm was condensed with concomitant reduction in nuclear size and degenerated RER and DCVs (Figure 7(b)). The ventral prostate epithelium of arecoline-treated rats showed an enlarged nucleus with abundance of well-organized RER (Figure 7(c)). Arecoline treatment of diabetic rats demonstrated ventral prostate epithelial cells with large euchromatic nucleus and abundance of SER (Figure 7(d)), which is almost similar to that of the control or arecoline-treated prostate cells of normal rats.  Table 3 summarizes quantification of the changes observed at the ultrastructural level.

### 3.9. Arecoline Increases the Expression of pdx-1 and GLUT-2 in Alloxan-Induced Diabetic Rats

**Figure 8:**
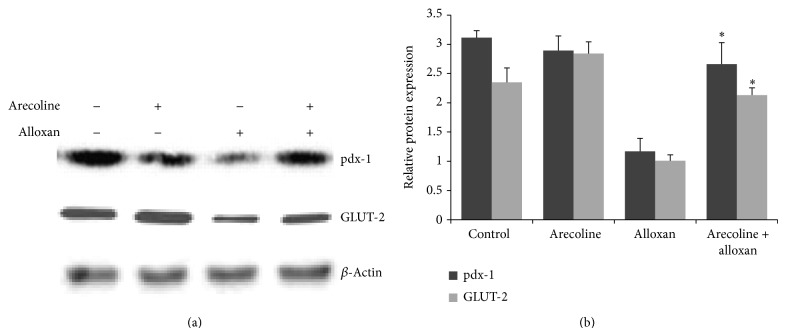
Changes in expression of proteins involved in islet regeneration and *β*-cell function. Diabetic rats were treated without or with arecoline. Protein expressions of GLUT-2 and pdx-1 were examined in cell lysates from the pancreas of the rats. Protein bands shown are a representative from three independent experiments with similar results; ^*^ *P* < 0.01.

*β*-cell-specific genes, including GLUT-2 and pdx-1, are critical for islet regeneration and *β*-cell function. We therefore assumed that changes in the expression of these genes might contribute to alloxan-induced reduction of serum insulin level. For this purpose, western blot analysis was performed to assess any changes in the expression of these genes. As shown in Figure 8, a significant decrease in protein expressions of pdx-1 and GLUT-2 was observed in alloxan-induced diabetic rats whereas, concomitant with the increase in insulin levels, considerable increase in the protein expressions of pdx-1 and GLUT-2 was detected in the pancreas after diabetic rats were treated with arecoline.

## 4. Discussion

Almost 600 million betel nut chewers are found worldwide [26]. It is the fourth most popular addiction for people in the South Pacific islands, Southeast Asia, Pakistan, and Bangladesh after tobacco, alcohol, and caffeine [27] and acts as a psychoactive drug [28]. The habit of chewing areca nut is endemic throughout the Indian subcontinent and the prevalence of areca nut use is rising in India and Taiwan [26]. It has been found that each chewer in Taiwan consumed approximately 14 to 23 betel quids a day [29]. Studies on the effects of betel nut chewing have confirmed that, amongst other effects, its consequences on the male reproductive physiology are of immense concern, since it elevates serum FSH, LH, and testosterone levels and leads to cellular changes that may alter the normal functioning of the prostate gland [13].

Since arecoline has a hypoglycemic effect [30] and betel nut chewing has been associated with higher risk of diabetes [1], the relevance of the effects of arecoline on serum insulin levels of experimentally induced diabetic rats and its consequences on the male steroid pathway and sex accessories was analyzed with the presumption that a sizeable proportion of the 600 million betel nut chewers may also be diabetic. Alloxan, a well-known diabetogenic drug, was used to induce a type-1 form of diabetes in rats, characterized by low insulin levels and hyperglycemia. Alloxan is known to lead to reproductive dysfunctions by decreasing the epithelial diameter, luminal volume, and stromal density of seminiferous tubules [31] and lowering the plasma testosterone concentration in rats [32]. In alloxan-induced diabetic rats, there is significant increase in the SER, mitochondria, and lipid contents of the Leydig cells [33]. These alterations may be attributed to the fact that alloxan inhibits antioxidants like superoxide dismutase and glutathione reductase activities in testis, along with significant elevation of testicular lipid peroxidation [34]. Additionally, alloxan significantly decreases glucose oxidation of ventral prostate in rats [35, 36]. In diabetic rats, the prostate shows an increase in number of cytoplasmic vacuoles with thickening of extracellular matrix [37] and decreased concentration of androgen receptors [36].

In agreement with previous reports, we found that alloxan significantly decreased blood insulin levels and consequently led to hyperglycemia in rats [38], since it is known to cause pancreatic *β*-cell damage, resulting in the reduction of insulin production in rats. Arecoline on the other hand is known to cause type 2 diabetes, characterized by insulin resistance [1]. Betel nut extract and arecoline also have diabetogenic potential on adipocytes that may result in insulin resistance and diabetes at least in part via the obstruction of insulin signaling and the blockage of lipid storage [3]. However, it has not been reported to cause type 1 diabetes till date. Also it is unlikely that arecoline would contribute to type 1 diabetes, since there is no evidence till date that arecoline has deleterious effects on pancreatic beta cells. Therefore we determined if this alkaloid could in any way ameliorate the levels of testosterone and insulin in alloxan-treated type 1 diabetes-induced rats.

Alloxan treatment caused suppression of Leydig cell activity with reduced testosterone level. Leydig cell degeneration was indicated by indented hyperchromatic nucleus and disorganized and dilated SER, which are indications of degenerative changes [39]. These degenerative changes of the Leydig cells correlated with the reduction in the serum testosterone level. Earlier, Kokk et al., 2007 [40], also reported that the reduction in testosterone levels was due to low LH levels in alloxan-induced diabetic rats [41]. Our results demonstrated that decrease in insulin levels led to decline in serum testosterone levels in diabetic rats. This conforms to the fact that insulin augments testicular androgen production by inhibiting sex hormone binding globulin (SHBG) concentration [42, 43]; therefore lower insulin should lead to decreased serum testosterone. Decreased testosterone production, on the other hand, inhibited development of male sex accessories, including growth of the prostate gland [44]. It is known that insulin receptors are located in the epithelial cells of the prostate gland [45]. Since epithelial cells of the prostate showed reduced nuclear size and disorganized mitochondrial cristae, they were rendered less responsive to the actions of insulin. In addition to the prostate gland, there was a fall in the fructose and sialic acid contents of the coagulating gland and seminal vesicle, respectively, possibly due to atrophy of the coagulating gland [46] and reduced weight and secretory activities of the seminal vesicles [47, 48]. Induction of diabetes with alloxan was also associated with decrease in hepatic glycogen, which could be attributed to the decrease in the availability of the active form of enzyme glycogen synthetase, probably because of low levels of insulin. In the present study, arecoline not only restored the depressed hepatic glycogen levels possibly by increasing the level of insulin, but also indicated effective glucose tolerance, as revealed by the IPGTT. Our results thus showed that supplementation of diabetic rats with arecoline resulted in significant elevation of hepatic glycogen content, which indirectly suggests the activation of glucagon, possibly as an additional consequence of increased levels of plasma testosterone.

We further explored a plausible mechanism by which arecoline reversed the levels of insulin and glucagon in diabetic rats, with special emphasis on how arecoline effectively overcame beta cell degeneration induced by alloxan and increased insulin production in type 1 diabetic rats. It is well established that the pancreatic duodenal homeobox-1 (pdx-1) is an orphan homeodomain transcription factor, which is normally expressed in *β*-cells and plays an important role in the development of the pancreas [49]. Although pdx-1 gene expression is generally not required for pancreatic determination of the endoderm, it is essential for the development of endocrine and exocrine cell types [50, 51]. Differentiation and maintenance of the *β*-cell phenotype also require pdx-1. In mice, *β*-cell-selective disruption of pdx-1 led to the development of diabetes with increasing age and was associated with reduced insulin and GLUT-2 (a glucose-sensing and -transporting molecule located on the surface of *β*-cells) expression [52]. Indeed, mice heterozygous for pdx-1 were found to be glucose intolerant [52]. In addition, impaired expression of pdx-1 as a consequence of hyperglycemia or increased lipid concentrations was thus associated with diabetes [53]. Our results conform to the above since alloxan treatment led to reduced expression of not only pdx-1 but also GLUT-2, since pdx-1 transcriptionally activates the gene encoding GLUT-2 [54]. Therefore, it can be strongly opined that when alloxan led to reduction in insulin levels and associated hyperglycemia by decreasing the expression of pdx-1 and GLUT-2, arecoline could overcome *β*-cell degeneration and effectively restored normal levels of hormones by increasing the expression of pdx-1 and GLUT-2. This finding thereby suggests that arecoline can be used to revert type 1 diabetes in rats.

It is thus interesting to note that arecoline treatment of alloxan-induced diabetic rats restored normal levels of hormones and eventually testicular function, whereby the following bona fide changes were observed: (i) Leydig cells showing enlarged nucleus with abundance of SER; (ii) increased production and secretion of testosterone and gonadotropins; (iii) structural integrity of the prostate being restored, as evident from the ultrastructure of the gland; and (iv) increased production of fructose and sialic acid content of the coagulating gland and seminal vesicle, respectively, as compared to that in diabetic rats. This recovery of hormonal, structural, and biochemical parameters related to male reproductive physiology may be attributed to the increase in serum insulin levels in arecoline-treated diabetic rats, by reverting pancreatic *β*-cell degeneration, and may thereby act as a positive protective factor for men with type 1 diabetes.

## Figures and Tables

**Table 1:** Effect of arecoline on liver glycogen content.

Parameter	Control	Arecoline	Alloxan	Arecoline + alloxan
Liver glycogen (ug/mg)	2.5 ± 0.08	2.14 ± 0.06	1.2 ± 0.07	2.4 ± 0.05^*^

Values are presented as mean ± SEM; *n* = 5 in each group; ^*^ *P* < 0.01.

**Table 2:** Quantitative changes in ultrastructural components of the Leydig cells of male rats.

Cell organelles	Control	Alloxan-induced diabetic rats	Healthy rats treated with arecoline	Diabetic rats treated with arecoline
Size of the nucleus (*μ*m)	12.61 ± 0.8	6.34 ± 1.2	16.41 ± 0.7	13.83 ± 0.6^*^
Number of DCVs	31.32 ± 0.08	11.23 ± 0.05	68.21 ± 0.06	42.12 ± 0.04^*^
Number of CVs	22.13 ± 0.04	9.13 ± 0.02	79.23 ± 0.04	20.12 ± 0.05^*^
Number of SER	29.14 ± 0.08	10.65 ± 0.08	64.23 ± 0.08	28.23 ± 0.08^*^

^*^ *P* < 0.01.

**Table 3:** Quantitative changes in ultrastructural components of the prostate epithelium of rats.

Cell organelles	Control	Alloxan-induced diabetic rats	Healthy rats treated with arecoline	Diabetic rats treated with arecoline
Size of the nucleus (*μ*m)	6.61 ± 0.7	2.34 ± 0.9	8.41 ± 0.4	5.83 ± 0.2^*^
Number of DCVs	11.32 ± 0.09	5.23 ± 0.05	18.21 ± 0.06	12.12 ± 0.04^*^
Number of SER	32.14 ± 0.51	8.65 ± 0.7	54.23 ± 0.8	34.23 ± 1.2^*^

^*^ *P* < 0.01.
